# Alcoholic Liquors in the Practice of Medicine

**Published:** 1887-07

**Authors:** Eugene Foster

**Affiliations:** Augusta, Georgia


THE ATLANTA MEDICAL AND SURGICAL JOURNAL.

VOL. IV. JULY, 1887. NO. 5.

### Original Communications.

### ALCOHOLIC LIQUORS IN THE PRACTICE OF MEDICINE.

READ BEFORE THE MEDICAL ASSOCIATION OF GEORGIA AT ITS THIRTY-EIGHTH ANNUAL SESSION IN ATLANTA, APRIL 21, 1887. BY EUGENE FOSTER, M. D., AUGUSTA, GEORGIA.

Never within the memory of the writer has so novel and important a question been forced upon the attention of the medical profession of Georgia as that of the present status of public opinion concerning "the relation of the medical profession to the use and abuse of alcoholic liquors."

Novel, in that a purely scientific question in medicine, which can only be intelligently and impartially investigated and decided by the medical profession, has been taken up, decided and acted upon by temperance crusaders, who have thereby usurped the office of the professors of the healing art. The temperance crusaders in this State have written voluminously, and in their writings have demonstrated their meagreness of knowledge on this subject; and while their writings and speeches have been most eloquently presented, they have reasoned from the most incorrect and inexact data, and thereby rendered their conclusions worthless in properly moulding public opinion, and injurious and burdensome to the sick and afflicted if adopted.

Important, in that the learning, integrity and fidelity of the medical profession have been most unwarrantably assailed by the enactment of the so-called "Local Option Bill," which met the approval of the recent Legislature of Georgia. Great, indeed, must be the urgency of the case where the people of a State or country dare take upon themselves the right to invade the domain of scientific medicine and say to its professors, here is a certain article which, notwithstanding we find it classified in your materia medica as a known and approved therapeutic agent, you shall not prescribe for the relief of human sickness and suffering, even though you propose to use it solely as a medicine. What warrant can these men offer for such unseemly and stupendous effrontery? None whatsoever. Their course cannot be justified either in equity or morals among a people who have not run mad with fanaticism. It is no part of my purpose to discuss the frightful evils of intemperance as a sociological problem. No man observes with more alarm and profound commiseration the baneful effects of alcoholic intemperance upon our people than I do. I have ever been, and am now, ready to join in any reasonable measure of legislation which shall remove temptations to the use of alcoholic liquors as a beverage or drink in common use among our people. I most heartily sympathize with the object of the temperance crusade—*i. e.*, the suppression of intemperance, but with all my heart and soul I denounce the means which they have resorted to so far as attempting to saddle the responsibility for any considerable proportion of intemperance upon the medical profession by the profession contending for the therapeutic value of alcoholic liquors. Every such assertion, or attempt to create such an impression, is absolutely and mischievously untrue. Likewise is every such statement that any large proportion of drunkenness is directly the outcome of the injudicious prescription of alcoholic liquors by medical men as a profession.

The only reason why these crusaders have dared to invade the temple of medicine, and brazenly denied us under heavy penalty the right to prescribe, and cause to be furnished to the sick, alcoholic liquors when deemed by us necessary, is the fact that a few, very few, learned and honorable physicians have expressed grave doubts as to the therapeutic value of these drugs. That these medical men were conscientious and sincere in believing their views to be correct, I have not a shade of a shadow of doubt. I am satisfied, however, that such opinions are founded upon incomplete or imperfect examinations into the question, and therefore that their conclusions were arrived at by most unscientific reasoning upon a scientific problem. In this paper I propose to examine, honestly and frankly, into the question of the value and uses of alcoholic liquors in medicine, and hope by patient and industrious research to place before the busy practitioner, in as concise a manner as justice to the intelligent presentation of the subject will allow, the scientific status of the question.

At the meeting of the Medical Association of Georgia in 1886, Dr. Joseph P. Logan, of Atlanta, one of the most learned, conscientious, honorable and highly-beloved physicians in this State, read a paper upon "The Relations of the Medical Profession to the Use and Abuse of Alcoholic Liquors." This paper has subsequently been published in THE ATLANTA MEDICAL AND SURGICAL JOURNAL, and more recently in the Atlanta *Constitution*, a daily newspaper in this State. Some of the views of Dr. Logan on the question are so novel, so at variance with all experience of the profession in all civilized countries, and while unintentionally so, so inexact as to be misleading and injurious, if not controverted, that I have, after much hesitation, and by urgent solicitation, decided to take up and examine some of the most important propositions enunciated by him.

In stating Dr. Logan's propositions, I shall use his language *verbatim*. I premise this examination by a reminder that I understand him to plant himself upon the text-writers in medicine, and I accept the issue. Wherever medical opinion is evoked, it is to be distinctly understood the opinions of text-writers, or standard authorities, are to govern. At this point I wish it to be distinctly understood that the review of Dr. Logan's paper relates solely to the scientific aspects of the question as to the value of alcoholic liquors in the practice of medicine. I shall refer to and discuss the local option bill, and the claims and purposes of the organized temperance movement in this State, but in so doing I insist that my language has no reference, either directly or indirectly, to Dr. Logan personally, or to anything contained in his essay. I yield to no man in my great respect for his high character and eminent professional attainments. With this preliminary statement, I proceed to examine the propositions to be discussed:

*Proposition 1.*—"Alcoholic liquors have no absolutely defined place in therapeutics."

Let us open our text-books and measure the correctness of this statement. The following standard books, embracing nearly all of the highest authorities on the subject known to the profession of medicine in America, state distinctly that alcoholic liquors, in moderate doses, stimulate the heart beneficially in disease:

Bartholow's Materia Medica and Therapeutics; Nothnagel and Rossbach's Materia Medica; Naphey's Modern Therapeutics; Headland on the Action of Medicines; Stille's Therapeutics and Materia Medica; Trousseau's Therapeutics; Wood (G. B.) Therapeutics and Pharmacology; Wood (H. C.) Therapeutics, Materia Medica and Toxicology; Biddle's Materia Medica; Farquharson's Guide to Therapeutics; Ringer's Handbook of Therapeutics; Carpenter (W. B.) on Use and Abuse of Alcoholic Liquors; Richardson (B. W.), Diseases of Modern Life; United States Dispensatory, last edition; Reynolds' System of Medicine; Fothergill's Handbook of Treatment; Flint's Practice of Medicine; Quain's Dictionary of Medicine; Bartholow's Practice of Medicine; Niemeyer's Text-book of Practical Medicine; Watson's Practice of Medicine; Bennett's Practice of Medicine; Ziemssen's Cyclopædia of Practical Medicine; Buck's Reference Handbook of Medicine; Pepper's System of American Medicine; Aitken's Handbook of Treatment; Bristowe's Theory and Practice of Medicine; Lionel Beale, author of "Bioplasm" and of "Protoplasm;" Robert's Handbook of Medicine; Lethely on Food; Pavy on Food and Dietetics; Wilson on Continued Fevers; Lyons on Fever; Anstie on Stimulants and Narcotics; Dupre on the Physiological Action of Alcohol; Beard on Stimulants and Narcotics; Parkes' Practical Hygiene; Ott on the Action of Medicines; Binz's Therapeutics; Hillier, Diseases of Children; Meigs and Pepper, Diseases of Children; Eustace Smith, Wasting Diseases of Children; Ellis, Diseases of Children; Vogel, Diseases of Children; West, Diseases of Children; Parker, the Puerperal Diseases; Rigby's System of Midwifery; Playfair's System of Midwifery; Churchhill's System of Midwifery; Barnes' (Fancourt) Manual of Midwifery; Ramsbotham's System of Obstetrics; Lusk, Science and Art of Midwifery; Brunton's Pharmacology, Therapeutics and Materia Medica; Ziemssen's Handbook of Therapeutics.

*Stimulant to the Nervous System.*—Without detaining you to quote the authorities, I state distinctly that almost every one, if not all, of the authorities cited in the preceding section state that alcoholic liquors, in moderate doses, stimulate beneficially the nervous system in disease.

*Alcoholic Liquors in Moderate Doses Promote Digestion.*—The following authorities affirm this proposition:

B. W. Richardson, Diseases of Modern Life; W. B. Carpenter, Use and Abuse of Alcoholic Liquors; Beard, Stimulants and Narcotics; Anstie, Stimulants and Narcotics; Bartholow's Materia Medica and Therapeutics; Quain's Dictionary of Medicine; Fothergill's Handbook of Treatment; Wood (G. B.), Therapeutics, Pharmacology and Materia Medica; Wood (H. C.), Therapeutics, Materia Medica and Toxicology; Ringer's Handbook of Therapeutics; Biddle's Materia Medica; Stille's Therapeutics and Materia Medica; Nothnagel and Rossbach's Materia Medica; Naphey's Modern Therapeutics; Routh, Infant Feeding; Trousseau's Therapeutics; United States Dispensatory, last edition; Pavy, Food and Dietetics; Lethely on Food; Chambers on Indigestion; Hartshorne, Essentials of Practical Medicine; Parkes, Practical Hygiene; Buck's Reference Handbook of the Medical Sciences; Farquharson, Guide to Therapeutics; Binz, Elements of

Therapeutics; Ellis, Diseases of Children; Smith, Wasting Diseases of Children; Loomis, Practical Medicine; Robert Handbook of Medicine; Bristowe, Theory and Practice of Medicine; Reynolds' System of Medicine; Flint's Practice of Medicine; Edward Smith on "Foods;" Brunton's Pharmacology, Therapeutics and Materia Medica.

This list might be extended to a great length, but as the intention of its presentation is to show accord of professional opinion on this subject, I will rest content with it as it stands.

*Proposition 2.*—"Alcoholic liquors cannot be proven to occupy any distinctive or legitimate position as agents for the treatment of disease."

Let us first examine the words distinctive and legitimate.

A distinctive position should properly and well be said to be one "well-marked, plain, obvious."

A legitimate position is one which is real, not false, fairly deducible, or, as defined by Webster, "according to, or authorized by, the laws or rules of a science or an art."

In order not to prolong this paper to improper length, I will merely say in reply that by the authorities just quoted I have shown that alcoholic liquors have, by general consensus of medical opinion, the following "well-marked," "plain," "real" positions as agents for the treatment of disease:

1. Cardiac stimulant.
2. Nervous stimulant.
3. Promoter of digestion.

*Proposition 3.*—"There are the most discordant, loose and undefined views in our approved medical works as to their application, and practically no exact therapeutic rules clearly established as to their *modus operandi* in special diseases, or as to the quantity in which they are to be administered, and thus lacking all recognized essential elements of approved and reliable medicinal agents."

This is certainly a novel proposition in the light of the teachings of "our approved medical works." Let us turn to typhoid fever, diphtheria, pneumonia and puerperal fever, and see what "views as to their application, what exact therapeutic rules are clearly established as to their *modus operandi* in these special diseases, or as to the quantity in which they are to be administered."

*Typhoid Fever.*—The following authorities agree substantially on the following proposition:

In a large proportion of cases no alcoholic liquors are necessary from first to last; it is scarcely ever required in the early stages of the disease, and at no period should it be given as a matter of routine, or merely because the case is one of fever, but only to meet certain definite indications. These are mainly evidences of weakness of the heart—frequent, weak and fluttering pulse, and weakness or absence of the first sound of the heart.

Quain's Dictionary of Medicine; Loomis' Practical Medicine; Reynolds' System of Medicine; Flint's Practice of Medicine; Niemeyer's Text-book of Practical Medicine; Trousseau's Clinical Medicine; Bartholow's Practice of Medicine; Wood's Practice of Medicine; Watson's Practice of Physic; Bennett's Practice of Medicine; Bristowe's Theory and Practice of Medicine; Robert Handbook of Medicine; Fothergill's Handbook of Treatment; Aitken's Handbook of Treatment; Ziemssen's Cyclopædia of the Practice of Medicine; Pepper's System of American Medicine; Buck's Reference Handbook of the Medical Sciences; Forbes and Tweedie's Cyclopædia of Practical Medicine; Lyons on Fevers; Bartholow's Materia Medica and Therapeutics; Stille's Therapeutics and Materia Medica; Biddle's Materia Medica; Wood (H. C., jr.), Materia Medica and Toxicology; Ringer, Handbook of Therapeutics; Nothnagel and Rossbach, Materia Medica; Trousseau, Therapeutics; United States Dispensatory; Anstie, Stimulants and Narcotics; Carpenter, W. B., on Alcoholic Liquors; Wilson, Continued Fevers; Hillier, Diseases of Children; Meigs and Pepper, Diseases of Children; Brunton's Pharmacology, Therapeutics and Materia Medica; Ellis, Diseases of Children; West, Diseases of Children; Ziemssen's Handbook of Therapeutics.

I will not detain you with the authorities advocating alcoholic liquors in pneumonia, diphtheria, puerperal fever, etc., where adynamic conditions complicate these diseases. In all adynamic conditions alcoholic liquors must be resorted to in some form temporarily to sustain life until the crisis is passed. Richardson, the celebrated temperance crusader, has about expressed the true status of the question when he said: "There are times in the life of man when the heart is oppressed, when the resistance to its motion is excessive, and when blood flows languidly to the centres of life, nervous and muscular. In these moments alcohol cheers. It lets loose the heart from its oppression; it lets flow a brisker current of blood into the failing organ; it aids nutritive changes, and altogether is of temporary service to man. So far alcohol may be good, and if its use could be limited to this one action, this one purpose, it would be amongst the most excellent of the gifts of science to mankind." This opinion is expressed by every text-writer but one of any standing as an authority in American medicine. I must confess that, after a thorough research of the authorities, I cannot endorse the statement that "there are the most discordant, loose and undefined views in our approved medical works as to their application, and practically no exact therapeutic rules clearly established as to their *modus operandi* in special diseases, or as to the quantity in which they are to be administered, and thus locking all recognized essential elements of approved and reliable medicinal agents." To the contrary I state that the text-books show accord of opinion, exactness and definiteness of views as to the application of alcoholic liquors in special diseases, and state the *modus operandi* of these compounds, and the dose thereof, in special diseases with as much definiteness and exactness as they do of almost any other approved and reliable medicinal agent. The *modus operandi* of any given medicine—even the best known—is a matter largely of speculation, and different text-writers hold diametrically opposite opinions in relation thereto, and yet these medicines are amongst our most approved and reliable medicinal agents. Take, for instance, quinine in the cure of malarial fevers. What do we know definitely as to the *modus operandi* of quinine in malarial diseases or any other disease? What of mercury in the cure of syphilis, or of opium in relieving pain? You or I may speculate upon the *modus operandi* of these medicines, but we know very little definitely of the matter. We know the blind, empirical fact that quinine cures malarial fever, mercury cures syphilis and opium relieves pain, and are content to prescribe these remedies, which are the most certain in results of any in the whole materia medica. The quotation just cited from Richardson states correctly the status of professional opinion as to their (alcoholics) application in disease, and their *modus operandi* in special diseases where adynamia is to be combatted. Now as to the objection that text-writers fail to agree upon the quantity or dose of each alcoholic liquor in use. Gentlemen, the objection is captious. There is absolutely no dose or quantity to be definitely stated of any medicine in the materia medica applicable to all cases or all persons alike. Stille truly says "the term dose, then, is a relative one; it designates a quantity which must vary with the peculiarities of the patient and the nature and stage of his disease." It is true that all medicines have what is designated as an officinal dose—which is the quantity found to be applicable to a majority of persons taking the drug—and all authorities agree as fully upon the doses of alcoholic liquors in disease as upon any other remedy in the materia medica.

*Proposition 4.*—"It may be stated with absolute certainty that it (alcohol) is antagonistic to all physiological operations, and furnishes no aid whatever to any vital process; but, on the contrary, the normal activities are diminished, as strikingly manifested by a depressed temperature and a decreased muscular power."

This, gentlemen, is a novel assertion to emanate from one who speaks from the books. We have already seen that to the contrary alcoholics in moderate doses aid both "physiological operations and vital processes," or to again use the cogent expression of Dr. B. W. Richardson, when speaking of diseases producing oppression of the heart, when the resistance to its motion is excessive, and when blood flows languidly to the centres of life, nervous and muscular, "In these moments alcohol lets loose the heart from its oppression; it lets flow a brisker current of blood into the failing organs; it aids nutritive changes." This opinion of Dr. Richardson is entertained and positively and clearly expressed by almost every text-writer known to American medicine. Indeed, I know of but one single text-writer of prominence who dissents from it—*i. e.*, Dr. N. S. Davis, of Chicago. Here, then, we see that it (alcohol) stimulates beneficially the heart and nervous system in disease and aids digestion, and yet we are informed and expected to concede "that it is absolutely certain that it (alcohol) is antagonistic to all physiological operations, and furnishes no aid whatever to any vital process." Gentlemen, if this assertion be true I ask for the proof. Certainly the proof cannot be found in the text-books. It may be that the maintenance of the circulation in its normal condition, and also the nervous system, and promoting digestion, are antagonistic to all physiological operations, but if so, physiology will have to be remodeled. It may be that an agent which maintains the blood circulation and nervous system in their normal condition and promotes digestion offers no aid to any vital process, but if so, circulation of blood and nerve force and digestion are not vital processes. But the most incorrect part of this paragraph is yet to be considered—*i. e.*, "but to the contrary the normal activities are diminished (by alcohol), as strikingly manifested by a depressed temperature and a decreased muscular power."

Now what do we know of its effects upon human temperature? It has been proven over and over again incontestably that alcohol does not lower the temperature directly unless in toxic doses, and only after profound intoxication does the decrease of temperature reach one or more degrees.

Let us briefly state what is definitely known of the effects of alcohol on the human temperature: 1. Moderate doses of alcohol produce in some persons in health a decrease of temperature amounting to from one-tenth to several tenths of one degree, Farenheit. In many persons moderate doses do not affect the temperature at all, while in yet another class the temperature is slightly elevated by moderate doses. 2. The lowering of temperature is altogether wanting in convalescents, or if at all diminished the diminution is markedly less than in the healthy subjects, the dose taken in each case being the same. 3. In those who habitually use alcoholic liquors the depression of temperature is almost always absent. 4. Frequent repetition of doses of alcoholics diminishes their lowering effect upon the bodily temperature. 5. The amount of diminution of temperature is directly proportionate to the dose of alcoholic stimulant taken. 6. The depression of temperature caused by alcoholic stimulants is of short duration, and the temperature soon regains the norme.

Now what do we know definitely as to alcoholic liquors producing decreased muscular power? Do we find muscular power invariably diminished by alcoholics, no matter what amount of stimulant has been taken? Unquestionably and unqualifiedly, no. Moderate doses of alcoholic stimulants do not decrease the power of the imbiber for sustained and strong muscular work. The amount of diminution of muscular power is directly proportional to the dose of alcoholic stimulant taken. As a usual thing no diminution of muscular power is observed in the adult who has taken 2 ounces of brandy or less, or its equivalent of other alcoholic liquors. I do not deny that alcoholic stimulants may produce all the evil effects set forth in the paragraph under review. Nay, I go further and say that alcoholic stimulants, when habitually used, are directly causative of a large percentage of dyspepsia, skin diseases, functional disturbances and degenerative changes of the heart and blood-vessels, the stomach, the lungs, liver, kidneys and nervous system. But whenever the drinking of alcoholic liquors be traced as the causal agency of either serious functional disturbances or organic diseases, such results will be found to follow the use of inebriant, toxic or lethal doses; and I assert positively and unqualifiedly that the judicious use of alcoholic stimulants for therapeutic purposes has never been known to be "antagonistic to any physiological operation nor to any vital process." Nor has any physician ever witnessed a depressed temperature (*i. e.*, greatly below the norme) nor a decreased muscular power from the judicious, therapeutic use of alcoholics in any form; and I assert the same in relation to the moderate use of alcoholic liquors as a beverage by persons in health. By moderate use of alcoholics I mean the occasional, though not habitual, imbibition of from one to four ounces of brandy, or its equivalent of other alcoholics, in the 24

hours. In these statements I am uncontradicted by any text-writer whose book is generally accepted as a text-book in American or European medical colleges.

It would certainly be reducing the argument to an absurdity to speak of the effects of alcoholics without specifying the kind used and the dose given. In other words, it is absurd to state that alcoholic liquors, in all forms and in all quantities, from a teaspoonful to a quart at a time, will prove antagonistic to all physiological operations and vital processes, and depress the temperature of the body, and decrease the muscular power of the individual; yet, gentlemen, this is exactly what almost all these temperance crusaders do. In doing this they ignore one of the cardinal laws of therapeutics—*i. e.*, the law of dosage. In the domain of therapeutics no law is more certainly fixed than this—*i. e.*, the action of a medicine varies with the dose or quantity in which it is given. In discussing the therapeutic uses of alcoholic stimulants, many temperance writers ignore this law entirely. They condemn alcoholics as pernicious, whether given by the teaspoonful or quart. To show the absurdity of attempting to exhibit the therapeutic value of a medicine while ignoring the dose, let us take an example:

Take tartar emetic. Give your patient several doses of one-twelfth of a grain, and you obtain an expectorant and diaphoretic action, with a reduction of the force of the pulse. Give him twelve grains at one time, and now observe the result. He is seized with nausea, violent vomiting, cramp in the stomach,feebleness of pulse, faintiness,burning in the œsophagus and stomach,vomiting mucus and bile and finally blood, purging from the bowels, the pulse becomes thready, the countenance pinched and livid, the skin cold and clammy, the temperature falls below the norme, with convulsions, stupor and finally, if not relieved, death from asthenia. Here we see what is the case with every medicine in the materia medica—*i. e.*, different effects according to the quantity or dose taken. I will not insult your intelligence by detailing the effects of alcoholics in quantities of one ounce and twelve ounces respectively. The man who would question this proposition should study the elemental principles of therapeutics.

*Proposition 5.*—"Indeed, I am strongly inclined to the conclusion that no greater delusion has been imposed upon the medical profession or the world than the idea that there is anything really beneficial in alcoholic liquors, either in health or disease, which cannot be substituted."

If this be true, and yet ninety-nine of every one hundred doctors in practice for the past one hundred years to the present time wholly ignorant of it, how instinctively we say, "What a wonderfully progressive profession is medicine!" But before condemning, let us turn over the pages of our "approved medical works," and seek the proof of the statement in question. It is singular indeed, yet the fact remains, that the essay under review entirely omitted the duty of furnishing us with the list of remedies which can be so successfully substituted for alcoholic liquors in disease. I ask now for the medicine which can be invariably substituted for alcoholic liquors as a promoter of digestion and stimulant to the heart and nervous system. Turning to "our approved medical works," I fail to find any writer of standing in "Materia Medica and Therapeutics" who even hints that the assertion above cited may be correct, much less do they confirm the statement. I have examined the following of "our approved medical works" and find them as silent as the grave when asked to substantiate Dr. Logan's views:

Ringer's Handbook of Therapeutics; Wood (H. C.), Materia Medica, Therapeutics and Toxicology; Brunton's Pharmacology, Therapeutics and Materia Medica; Farquharson's Therapeutics and Materia Medica; Binz, Elements of Therapeutics; Nothnagel and Rossbach, Materia Medica; Trousseau's Therapeutics; Biddle's Materia Medica; Bartholow's Therapeutics; Stille's Therapeutics and Materia Medica; Wood (G. B.), Therapeutics and Pharmacology; Headland on Action of Medicine; Naphey's Modern Therapeutics; Anstie, Stimulants and Narcotics; Beard (G. M.), Stimulants and Narcotics; U. S. Dispensatory; Brunton, Pharmacology, Therapeutics and Materia Medica; Ziemssen's Handbook of Therapeutics; Ott on the Action of Medicine.

To the same purpose I present the following standard authorities on practice of medicine, embracing almost the whole list of the highest and best modern authorities in practice of physic from all civilized countries known to American medicine:

Reynolds' System of Medicine; Loomis' Practical Medicine; Quain's Dictionary of Medicine; Flint's Practice of Medicine; Trousseau's Clinical Medicine; Bartholow's Practice of Medicine; Niemeyer's Text-book of Practical Medicine; Watson's Practice of Physic; Wood's Practice of Medicine; Aitken's Handbook of Treatment; Hartshorne's Essentials of Practical Medicine; Ziemssen's Cyclopædia of Practice of Medicine; Robert's Handbook of Medicine; Bristowe's Theory and Practice of Medicine; Pepper's System of Medicine; Buck's Reference Handbook of the Medical Sciences; Bennett's Practice of Medicine.

Of all these authorities not one takes such views as Dr. Logan has enunciated. To the contrary, every one of them states that alcoholic liquors are indispensable in many diseases. None of them claim that alcohol is a universal stimulant—a panacea in all diseases to which human flesh is heir. There are times and cases where other stimulants can better serve the purpose of stimulant than alcohol, and all these authorities recognize and admit this fact. But no single one of them looks upon alcohol as a delusion and a snare in scientific medicine. The only book-writer known to me who takes any such view is Dr. N. S. Davis, of Chicago, Illinois. Dr. Davis claims that "from first to last alcohol acts as a paralyzant and an anæsthetic, and is in no sense a stimulant." He denies and ignores the therapeutic value of alcoholic liquors and acts as follows:

"In the prostration and low delirium of typhoid fever, he relies upon careful feeding and the use of strychnia and nitric acid."

"In the heart-failure, which he does not look upon as induced so much by the fever as by the defective oxygenation, he finds the stimulating effects of quinine, digitalis and chlorate of potash, with coffee, the most valuable remedies, which have succeeded in his hands when alcohol has failed."

I have no desire nor intention to speak lightly or disrespectfully of Dr. Davis, yet truth requires the statement that he holds novel opinions upon questions other than alcoholic liquors. The best way to test the opinion of the medical profession upon Dr.

Davis' book is to look to our medical colleges and ascertain how many of them recommend it to their students as a text-book on practice of medicine. I have written to every one of the best regular colleges in America, and find that of the whole number of catalogues received only two have Dr. Davis book on their catalogues.

But suppose every medical college in America recommended this book, I ask you, in all fairness, would it not be presumptuous indeed to attempt to make the naked negative statement of Dr. Davis outweigh the positive assertions and experiences of practically all the best text-writers on practice of medicine in England, France, Germany and America?

(*To be Continued.*)

